# Study protocol for the Breathe Easy All Together e-cigarette prevention program: a RE-AIM framework-based experimental process and impact evaluation

**DOI:** 10.1186/s12889-026-27623-7

**Published:** 2026-05-13

**Authors:** Catriona Lockett, Smita Shah, Kym Rizzo Liu, Emily Stockings, Matthew Sunderland, Julie Mooney-Somers

**Affiliations:** 1 Prevention Education and Research Unit, Research and Education Network, Western Sydney Local Health District, Sydney, NSW Australia; 2 Sydney School of Public Health, Faculty of Medicine and Health, The University of Sydney, Sydney, NSW Australia; 3 The Matilda Centre for Research in Mental Health and Substance Use, The University of Sydney, Sydney, Australia

**Keywords:** E-cigarettes, Vaping, Tobacco, Adolescence, Prevention, School-based intervention, Peer-Education/Peer-led

## Abstract

**Background:**

Effective preventative interventions are urgently needed to address the increasing rates of e-cigarette use among young people and to complement the Australian government’s latest tobacco control measures. School-based interventions using peer leaders can effectively shape social norms and expectations associated with tobacco use. This paper describes the protocol for the Breathe Easy All Together (BEAT) study, a process and impact evaluation of the first school-based peer-leadership intervention to prevent adolescent e-cigarette use in Australia.

**Methods:**

A pre-post test design will be used to conduct process and impact evaluation of the BEAT program using the RE-AIM (Reach, Effectiveness, Adoption, Implementation and Maintenance) framework. The intervention utilises a multi-component peer-education model driven by students, to promote respiratory health and resist e-cigarette use within a supportive school environment. Volunteer Year 10 students (14- to 16-years-old) will be trained by the research team as peer leaders, to deliver two 60-min lessons to Year 7 students (11- to 13-years-old) during routine health education classes. Year 10 peer leaders and Year 7 students will complete online self-report surveys pre- and post-program to assess changes in the primary outcome of past-30-day e-cigarette use. Secondary outcomes include tobacco use behaviours, e-cigarette knowledge, perceptions, refusal skills, intentions to use e-cigarettes, and acceptability of intervention components. School staff involved in the delivery of the program will participate in semi-structured interviews to examine acceptability and factors affecting program implementation. Descriptive statistics and changes between data collection points will be assessed using Wilcoxon signed rank tests to investigate intervention effect on primary and secondary outcomes. Qualitative data will be analysed in alignment with RE-AIM constructs using Framework Analysis.

**Discussion:**

Building upon an evidence-based peer-education model, BEAT presents a novel peer-led solution to address a gap in current research practice to prevent adolescent e-cigarette use in Australia. It has the potential to protect the future health and wellbeing of students from the harms of e-cigarette use and nicotine addiction. The RE-AIM framework is a comprehensive approach to conducting robust evaluation of multi-faceted interventions in the school environment. If effective, funding support will be sought for wider dissemination and further evaluation.

## Background

In recent years, the use of e-cigarettes (or ‘vaping’) has rapidly increased among young people, particularly among those who do not smoke. Global trends indicate the prevalence of e-cigarette ever use among young people (aged < 20 years) to be over 40% in high income countries, including the United States, France, Italy and Spain [1]. In a national survey of current smoking and vaping patterns across Australia, past 30-day e-cigarette use in adolescents aged 14—17 years has increased from 0.8% in 2018 to 14.5% in 2023 [2]. This rapid increase in adolescent e-cigarette use has been fuelled by several factors, including appealing flavours, social pressures, curiosity, targeted industry marketing, and misperceptions of e-cigarette use as safer than smoking [3].

Although e-cigarettes were originally designed as safer nicotine delivery systems for adult smokers, disposable e-cigarettes have gained popularity among young people who have never smoked [4, 5]. While the evidence for the health effects of e-cigarette use is limited and varies in strength, Banks et al.’s systematic review has shown e-cigarette use causes a range of acute cardiac and respiratory problems, including e-cigarette or vaping use-associated lung injury (EVALI), seizures, poisoning, burns and nicotine addiction [6]. Nicotine use in adolescence can lead to impaired brain development and worsened mental health outcomes, such as depression and substance abuse disorders [7]. Importantly, young people who use e-cigarettes are three times more likely to initiate tobacco smoking compared to those who do not use e-cigarettes [8].

In Australia, the purchase and sale of nicotine e-cigarette products to people aged under 18 years is illegal [9]. Despite this, regulatory gaps prior to 2024 have allowed nicotine e-cigarette products to become easily accessible to young people through retailers, tobacconists, convenience store, social media, and friends and family [5]. In response, in July 2024, the Australian Federal Government has implemented a suite of rapidly evolving regulatory and non-regulatory measures to quash the black-market and protect young people from the health risks of e-cigarette use [9]. While these regulatory changes predominantly focus on restricting the supply of e-cigarettes, other measures include significant investment in lung cancer screening, Tackling Indigenous Smoking, public health information campaigns, and smoking and vaping cessation support [10]. These measures will likely take months to years to have a broader effect on the e-cigarette market in Australia, and in the interim, e-cigarettes remain readily accessible and frequently used among young people. Thus it is critical that preventative interventions addressing the demand for tobacco and e-cigarette products are also implemented to empower young people with the knowledge, skills and confidence to resist and make informed choices about e-cigarette use [11]. Empowering young people may also motivate them to comply with the latest e-cigarette laws [12].

Schools are ideal settings for the delivery of preventative interventions as they provide access to a significant proportion of the adolescent population and can influence the establishment of health behaviours [13, 14]. Additionally, substance use prevention is a mandatory component of the personal development and health education curriculum in Australia [15]. Preventative interventions delivered in group or classroom settings can help adolescents make informed decisions about tobacco use while developing both self and collective efficacy [3, 16]. While there is mixed evidence for the effectiveness of school-based tobacco prevention programs, a systemic review and meta-analysis demonstrated strong evidence for multi-component interventions which integrate a combination of social competence and influence curricula [17]. Such programs have demonstrated a 12% reduction in the uptake of smoking compared to controls at 1 year follow-up [17]. Other components of successful tobacco prevention programs include the delivery of multiple sessions, interactive curricula, refusal skill practice, normative education, and use of peer leaders [3, 18, 19].

While numerous school-based e-cigarette prevention programs have emerged in recent years, informed by best practices in tobacco control, there is a paucity of evidence for their effectiveness. A 2024 systematic review of e-cigarette preventative interventions targeting children and adolescents found school-based interventions show mixed results in reducing the uptake, prevalence or intentions to use e-cigarettes [20]. Of 14 school-based interventions, only Above The Influence of Vaping (ATI-V) and INCLUSIVE were found to reduce the prevalence of e-cigarette use [21, 22]. One of two studies evaluating CATCH My Breath (CMB) reported a lower uptake of e-cigarette use compared to controls [23]. These interventions were aligned with best practices, delivering normative education with resistance and social skills training [20]. CMB and ATI-V also incorporated elements of peer-led programming [22, 23]. Most school-based programs effectively increased students’ knowledge and harm perceptions of e-cigarettes post intervention, although they produced no effect, increased, or decreased students’ intentions to use e-cigarettes [20]. Further research utilising robust evaluation methods is urgently required to identify effective components of e-cigarette prevention programs and the necessary conditions for successful implementation in the school context [3].

A systematic review of interventions aimed at children and adolescents [18] did not include any Australian interventions. There is currently one school-based eHealth e-cigarette prevention program, OurFutures, being evaluated in clustered randomised control trial in 42 secondary schools across Australia, with results still pending [24]. A range of effective, acceptable, and relevant school-based interventions are needed to complement government efforts to address increasing rates of e-cigarette use among young Australians.

In early 2021, the Prevention Education and Research Unit (PERU), Western Sydney Local Health District, initiated a community-led approach in response to local secondary schools’ appeals for help to address increasing rates of e-cigarette use among students, and the school-disruption associated with managing e-cigarette use. This approach is conducted in partnership with the New South Wales (NSW) Department of Education and Eastern Creek Principals Network with the aim to build the agency of school communities to reduce the initiation and use of e-cigarettes in students. Prior to the development or implementation of interventions to address student e-cigarette use, PERU conducted formative research to determine students, school staff, and parents’ perceptions of e-cigarette use and their preferred prevention strategies [25]. Informed by the findings this qualitative exploratory study, PERU developed the Breathe Easy All Together (BEAT) peer-leadership program, an adaptation of the Adolescent Asthma Action (Triple A) program, which has shown to be effective in improving asthma outcomes and resilience to tobacco initiation in Australia and internationally [26, 27]. BEAT complements the school curriculum and seeks to add value to the school community by providing students with an opportunity to be involved in the delivery of prevention messaging.

This research study seeks to conduct process and impact evaluation of the BEAT program, informed by the RE-AIM framework (reach, effectiveness, adoption, implementation and maintenance). The objectives of the study are to:

1. determine the impact of the BEAT program on Year 7 and Year 10 students’ e-cigarette use, combustible cigarette use, intentions to use, knowledge, perceptions and refusal skills.
2. explore the experience of Year 10 students as BEAT Peer Leaders in delivering the BEAT program.
3. describe program reach and adoption across participating sites.
4. identify barriers and facilitators affecting the implementation and ongoing maintenance of the BEAT program in school settings.

## Methods

### Participants

Eligible participants will be Year 10 students (aged 14—16 years) and Year 7 students (aged 11—13 years) attending participating secondary schools in NSW, Australia, between 2024 and 2025. Students may participate regardless of their smoking or e-cigarette use status. All students who do not opt-out of the study or are not opted-out by their parent/guardian, will automatically be entered into the study. School staff who are involved in the implementation of the BEAT program, including the BEAT program coordinator, will be eligible to participate. Schools receiving other e-cigarette prevention programs or are enrolled in trials testing such interventions (e.g. OurFutures and Reality Now) will be excluded.

### Intervention

The Breathe Easy All Together (BEAT) program is a multi-component peer-leadership program which aims to empower students by providing them with the knowledge, skills and confidence to make informed decisions about e-cigarette use and advocate for respiratory health (see Table 1). The BEAT program utilises an evidence-based peer-education model, driven by students, to promote respiratory health and resilience to e-cigarette use in a supportive school environment [27–29]. This model is underpinned by social cognitive theory and the empowerment education approach [30, 31]. Social Cognitive Theory centres on the concept of reciprocal determinism, where cognitive, behavioural and environmental factors interact to determine individual behaviour change [30]. The Empowerment Education Framework is an effective preventative health model in which people are empowered to take control over their lives as agents of social action within their communities [31]. Through education, participants are facilitated to determine their own priorities and the necessary actions to collectively affect environmental conditions. BEAT combines these methodologies to contribute to the creation of a supportive school environment in which students drive behavioural change through the delivery of normative education, peer role modelling, and social reinforcement.

**Table 1:** Overview of the BEAT Program content

Intervention component	Activities and key messages
Online Course	The online course provides normative education on: Chemical contents of e-cigarette, consequences of e-cigarette use, reasons why young people do and do not use e-cigarettes, alternatives to e-cigarette use and industry marketing tricks and tactics
Peer leader workshop for Year 10 students	The students participate in a series of interactive activities based on empowerment education which include: Group Agreements, asthma first aid, empowerment activity, unpacking tobacco statements, peer pressure to use e-cigarettes, refusal skill practice, life hack lotto highlighting alternatives to e-cigarette use, coping skills race, unpacking an image and industry marketing tactics and BEAT Peer Leader practice
Peer-education lessons for Year 7 students	These include: Group Agreements, online course, empowerment activity, peer pressure and e-cigarette refusal skill practice and life hack lotto highlighting alternatives to e-cigarette use

**Fig. 1:**
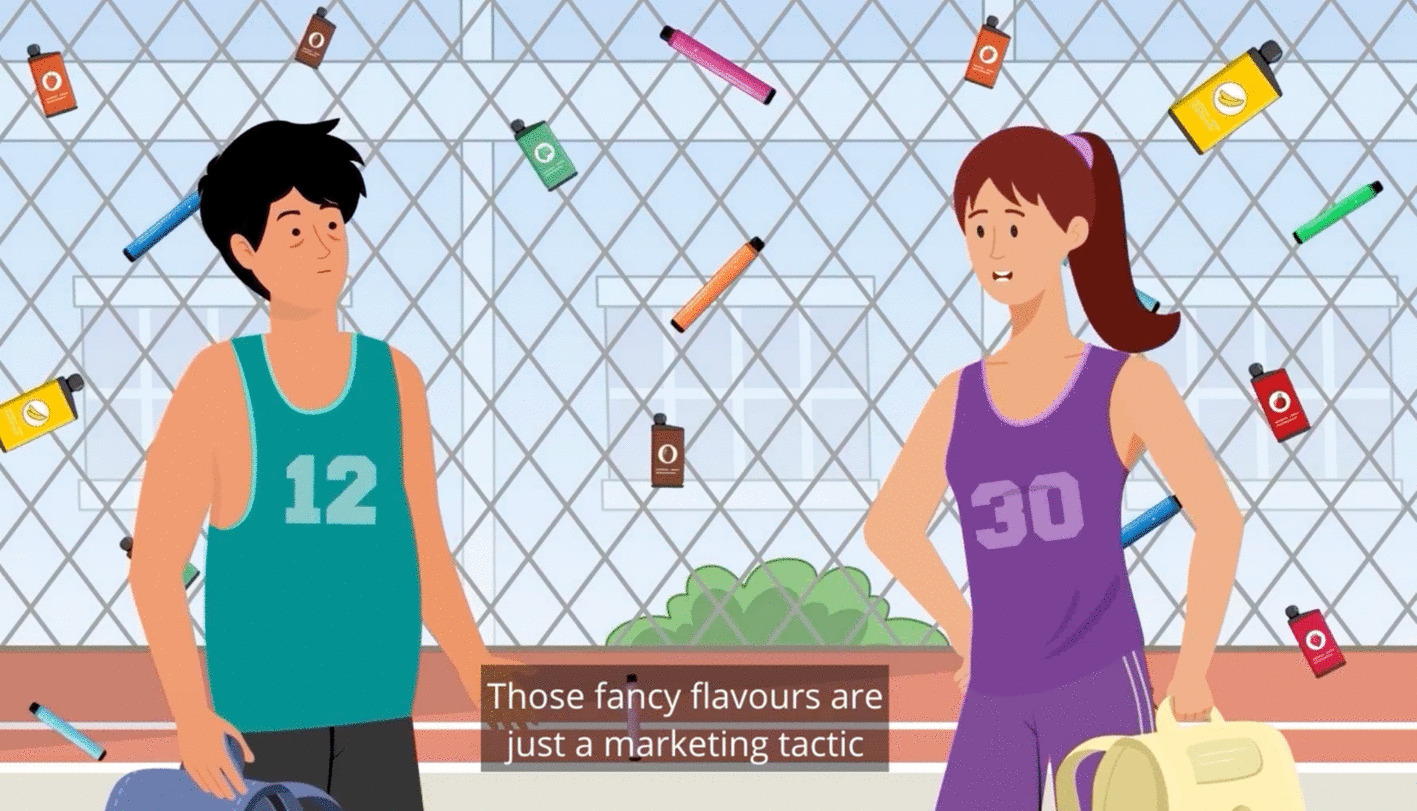
Example of video shown during the empowerment activity for Year 10 and 7 students

Steps of the BEAT Program:

1. Online Course: Year 10 students (approximately 15—30 students per school) who have volunteered or been selected to participate in the BEAT program as a peer leadership opportunity, complete a 45-min online course. The online course developed by PERU, provides students with normative education regarding the contents of e-cigarettes, harms of use, alternatives to use, and industry marketing strategies.
2. Peer Leader Workshop: The same group of Year 10 students participate in a one-day workshop held by the research team during school hours, where they receive education and engage in interactive activities to learn about asthma and what to do in an asthma emergency, resisting e-cigarette use, and acquiring skills in group facilitation (see Fig. 1). They practise being a BEAT Peer Leader, using a manual detailing the content of the peer-education lessons for Year 7 students.
3. Peer Education Lessons: BEAT Peer Leaders, in teams of four students, deliver 2 × 60-min lessons to Year 7 students during routine personal development and health education. The lessons include facilitation of the online course, followed by interactive activities and a game to reinforce learning. A classroom teacher will be present to assist with classroom management, and a member of the research team will be present to monitor intervention fidelity.

### Pilot testing

The BEAT program was piloted in four public secondary schools with 90 students to ensure its feasibility and acceptability. The formative evaluation was underpinned by an Action Research approach [32] which involved student surveys, group discussions, input from school staff, and observational notes. Modifications were made based on the feedback, addressing technical difficulties, adding group discussion opportunities, including physical activity and removing unpopular activities like pledges to remain e-cigarette free. Schools indicated a preference for a ‘flexible implementation’ approach to ensure alignment with school priorities which would allow schools to implement the 2 × 60 min peer-education lessons back-to-back or over the course of two weeks.

### Study design

This is a pre-post study seeking to change perceptions and behaviours of Year 7 and 10 students, with outcomes assessed both quantitatively and qualitatively. Students will complete surveys prior to and four-weeks after baseline to evaluate changes in knowledge, perceptions, refusal skills and intentions to use e-cigarettes. Mixed methods research generates rich insights about the conditions required for the successful adoption, implementation and maintenance of interventions [33]. The study has received ethics approval from the University of Sydney Human Research Ethics Committee (2024/HE000200).

### Sample size

This study aims to evaluate the effect of the BEAT program on knowledge, attitudes towards, and usage of e-cigarettes. The primary outcome variable measures the within subject change from pre- to post-intervention for students past 30-day e-cigarette use. At least 12 schools will participate with each school considered as a ‘cluster’. Within each school two cohorts of students, namely ‘Year 10 Peer Leaders’ and ‘Year 7 students’ will be studied. Based on past experience, it is expected that at least 15 Peer leaders and 100 Year 7 students at each school will complete the questionnaire both pre- and post-intervention.

### Power analysis

Each cohort will be considered separately. In order to maintain an overall 5% two-sided significance level, the null hypothesis of no change for the primary outcome variable across two time points will be tested using a paired Wilcoxon signed-rank test accounting for potential non-normality of the outcome. Given the naturally clustered data (e.g., students clustered within schools), the smallest detectable effect size is based on the “effective sample size” that was calculated by adjusting the proposed sample size by the study design effect. Assuming cohort sample sizes of 180 ‘Year 10 Peer Leaders’ (15 leaders per school) and 1200 ‘Year 7 students’ (100 students per schools), the study will have at least 80% power to detect the effect sizes for various intraclass correlation coefficients (ICC) ranging from 0.01 to 0.05. The minimal detectable effect sizes for the Year 10 Peer Leaders range between 0.230—0.281, whereas the minimal detectable effect sizes for the year 7 students range between 0.117—0.203 (see Table 2). All power calculations were conducted using G*Power 3.1.9.7.

**Table 2:** Range of detectable effect sizes in students

ICC	Cohort							
	Year 10 peer leaders (*n* = 12*15 = 180)				Year 7 students (*n* = 12*100 = 1200)			
	**m**	**DE**	**n/DE**	**Effect size required#**	**m = 100**	**DE**	**n/DE**	**Effect size required#**
0.01	15	1.14	158	0.230	100	1.99	603	0.117
0.02	15	1.28	141	0.243	100	2.98	403	0.143
0.03	15	1.42	127	0.256	100	3.97	302	0.166
0.04	15	1.56	115	0.270	100	4.96	242	0.185
0.05	15	1.7	106	0.281	100	5.95	202	0.203

^*#*^* Smallest Effect size detectable with 80% power*

## Procedure

### Setting

This study will be embedded within the existing health promotion services provided by PERU which primarily delivers school-based prevention programs to secondary schools within the greater western Sydney (GWS) area. GWS is one of the fastest growing and culturally diverse metropolitan populations in Australia, comprising 2.5 million people across 9,000 square kilometres [34]. It is characterised by a high proportion of newly settled migrants, those who speak a language other than English at home, and Indigenous Australians. Additionally, there is a higher proportion of low-income families, unemployment, and students who do not complete high school compared to the rest of Sydney.

### Recruitment

We have established longstanding relationships with secondary schools across western Sydney through the provision of health promotion initiatives and school-based prevention programs. Additionally, this study is delivered in partnership with the NSW Department of Education and secondary schools which have been involved in PERU’s ongoing community-led approach to address adolescent e-cigarette use. Schools involved in these networks will be invited to participate in the study. Schools outside of this network and which have previously expressed interest in the research will also be invited to participate. Due to capacity constraints of the research unit, precedence will be given to secondary schools within the GWS catchment.

**Fig. 2:**
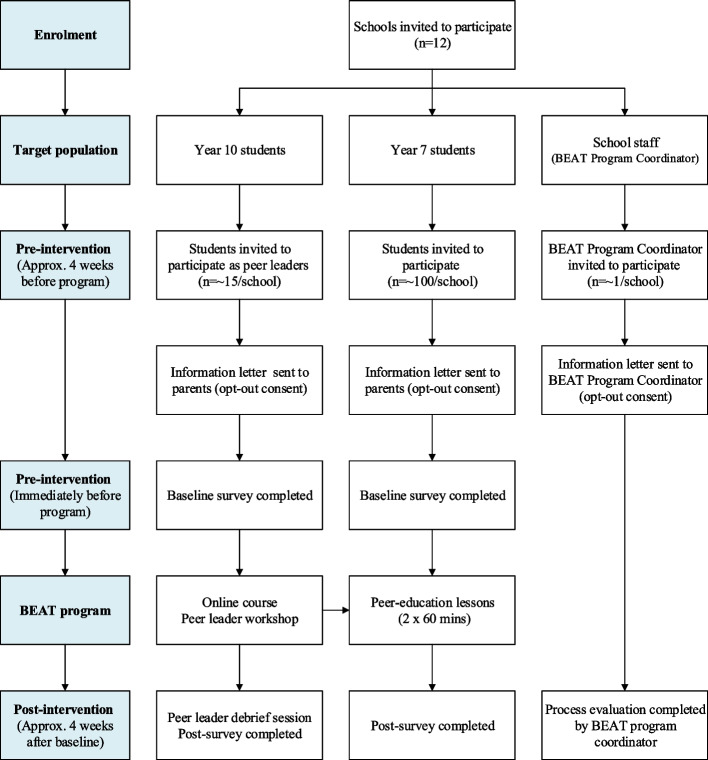
Anticipated recruitment and assessment of participants

An invitation will be sent to school principals outlining the study aims and seeking permission to implement the study. The research unit will follow-up schools via face-to-face visits and email. After the school principal has agreed to participate in the study, the school staff member nominated as the BEAT program coordinator will put out an expression of interest or select a group of Year 10 students to be trained as peer leaders. By using a flexible implementation approach, schools have the autonomy to recruit the Year 10 peer leaders according to their leadership frameworks. As part of seeking ethics approval, the process of engaging schools and obtaining their support for study was initiated early in 2024. Therefore, we anticipate that formal recruitment will take less than four weeks. Figure 2 provides an outline of the anticipated recruitment and assessment process of participants.

### Informed consent

This study will use an opt-out consent process for students. Participant information statements (PIS) will be distributed to parents/guardians and students via hardcopy or electronically at least two weeks before study commencement. The PIS for parents of Year 7 and 10 students includes information about how to opt-out by returning a signed form, if they do not wish for their child to participate in the study.

The school staff member responsible for coordinating the BEAT program within the school will electronically send an information letter to the parents of Year 7 and 10 students at least two weeks before program implementation. Additionally, the BEAT Program Coordinator will distribute a hardcopy or softcopy of the PIS participating students at least two weeks before program implementation and ensure students have read and understood the statement. Students are provided multiple opportunities to opt-out of the study: they can ask their parents to opt-out on their behalf or they may opt-out by speaking to the BEAT program coordinator or classroom teacher at any time before or during the study. At the beginning of the program, the research team will explain that students may leave the study at any point by speaking to the teacher or member of the research team present. Students will not be disadvantaged in anyway by opting out of the study and will have access to alternative learning arrangements in line with regular personal development and health education lessons. School staff involved in the program as the BEAT Program Coordinator will be asked if they have read and understood the PIS and requested to participate in a brief semi-structured interview.

### Assessments

Year 10 students trained as BEAT Peer Leaders and all Year 7 students that participate in the BEAT program will be invited to complete an online self-report survey in a supervised classroom setting at baseline immediately before the intervention and four-weeks after baseline. Hardcopy surveys will be available to schools during data collection, with data entered and checked by a member of the research team. Students absent for follow-up surveys will be contacted by the BEAT program coordinator via email and invited to complete the survey outside of school time. All data will be de-identified and linked using a personal code created by the research team.

## Measures

### Evaluation framework

The evaluation of BEAT is informed by the RE-AIM framework developed by Glasgow et al. [35] which includes five dimensions: Reach, Effectiveness, Adoption, Implementation and Maintenance (see Table 3) The RE-AIM framework is particularly advantageous for comprehensively evaluating complex settings such as the school environment as it bolsters the transparent and systematic reporting of intervention results considering both internal and external validity constructs [36, 37]. Such reporting enhances understanding of the generalisability of intervention results and the feasibility to broader implementation in “real-world” settings. The RE-AIM framework has been effectively used in studies evaluating school-based interventions targeting physical activity, nutrition, and community-wide approaches [38–40].

**Table 3:** RE-AIM evaluation dimensions and how these are quantified in the proposed study

RE-AIM Dimension (Level)	Definition of Assessment Criteria	Measurement Instrument or Process	Key Measures
Reach (Individual)	Number, proportion, and characteristics of participating students	• Pre-post survey administered to Year 7 and 10 students • Observation notes	• Student demographics • Participant rates in the intervention • Recruitment of peer leaders
Effectiveness (Individual)	Effect of intervention on the primary and secondary outcomes among Year 7 and Year 10 students	• Pre-post survey administered to Year 7 and 10 students	• Primary outcome: Past 30-day e-cigarette use • Secondary outcomes: use of nicotine products, intentions to use, knowledge, perceptions and refusal skills
	Year 10 students’ experiences as BEAT Peer Leaders	• Pre-post survey administered to Year 10 students • Debrief sessions • Semi-structured interview with BEAT Program Coordinator	• Student ratings of the program and feedback • Self-perceived gains • Perceived benefits for peer leaders
Adoption (Organisational)	Number, proportion and characteristics of participating schools	• Observation notes • Semi-structured interview with BEAT Program Coordinator	• Recruitment of schools • School type and ICSEA • Engagement with the program • Implementation of other e-cigarette related initiatives
Implementation (Individual and organisational)	Fidelity and quality of intervention delivery	• Observation notes	• Intervention exposure, adherence, and student responsiveness during peer leader workshops and selected year 7 lessons
	Barriers and facilitators to implementation	• Semi-structured interview with BEAT Program Coordinator • Debrief sessions	• Factors influencing delivery • Aspects that worked well, challenges encountered, suggested improvements
	Student responsiveness to the intervention	• Pre-post survey administered to Year 7 and 10 students	• Rating of intervention components (i.e., activities liked or not liked)
Maintenance (Organisational)	Extent to which the program is sustained over time within schools	• Observation notes	• Ongoing school engagement • Continued participation in research unit initiatives • Program refinement and adaptation

### Reach

For Year 10 students, reach is assessed by the number of students who complete the peer leadership training and deliver peer-education lessons to Year 7 students, compared to the number of students who initially volunteered to participate in the program. For Year 7 students, reach is assessed by the number of students who participate in both BEAT peer-education lessons compared to the enrolled number of Year 7 students in the school. Representativeness of students is assessed by comparing the baseline information reported by the participants, including demographic characteristics and tobacco use behaviours, with information obtained from population surveys of this group in NSW, Australia. Demographic measures include information on students’ year group, gender, Aboriginal or Torres Strait Islander identity, and language spoken at home.

### Effectiveness

#### Primary outcome

The primary outcome is change in past-30-day e-cigarette use reported among students. This will be assessed by asking ‘During the last 30 days, on about how many days did you vape?’ with response choices ranging from 0 to 30 days. The primary endpoint will be assessed at four weeks from baseline, post-intervention.

#### Secondary outcomes

##### **Tobacco use experience**

Measured by 10 questions adapted from the Youth Risk Behaviour Survey [41], validated measures of susceptibility [42, 43], and previous studies [7, 23, 44]. Participants are asked about past-30 day and ever use of tobacco products, including e-cigarettes and cigarettes. Response choices are Yes/No. Participants are asked how many of your close friends and family members currently use tobacco products. Responses choices include ‘none of them’, ‘some of them’, ‘most of them’, and ‘all of them’. Participants are also asked how easy or hard it would be to obtain a vape. Response choices on are on 5-point Likert scale, where 1 = ‘very easy’ and 5 = ‘very hard’.

##### **Asthma knowledge**

Measured by 6 questions adapted from previous surveys administered in young people aged 7—to—17 years [45, 46]. Year 10 students are asked questions relating to asthma symptoms, triggers and management. Scores will be calculated as a percentage of correct answers. Response choices are true/false/unsure.

##### **E-cigarette knowledge**

Measured by 6 questions developed to reflect the of content the BEAT program. These questions were piloted with students to ensure understanding and an appropriate level of difficulty. All students are asked about e-cigarette ingredients, health risks, legality, and industry marketing. Scores will be calculated as a percentage of correct answers. Response choices are true/false/unsure.

##### **Intent to try cigarettes and e-cigarettes**

Measured by four questions adapted from validated measures of susceptibility [42, 43] and previous studies [23, 44, 47]. Participants are asked about the likelihood of trying an e-cigarette or cigarette and willingness to use them if offered by a friend. Responses will be on a 4-point Likert scale, where 1 = ‘definitely not’ and 4 = ‘definitely yes’.

##### **Perceived harms and benefits of e-cigarette use**

Measured by 14 questions using an adapted and validated version of the Smoking Expectancy Scale for Adolescents [48, 49], and previous studies [23, 44, 47, 50]. Participants are asked about their agreement with seven statements regarding subjective norms and outcome expectations of e-cigarette use (i.e., “vaping can help reduce stress”). Response choices are on a 4-point Likert scale, where 1 = ‘strongly agree’ and 4 = ‘strongly disagree’. Participants are asked about the likelihood of five additional outcomes of e-cigarettes use (i.e. “your sports performance will get worse”). Response choices are on a 5-point Likert scale, where 1 = ‘not at likely’ and 5 = ‘extremely likely’. Participants are asked how harmful they perceive daily use of cigarettes and e-cigarette to be. Response choices are on a 5-point Likert scale, where 1 = ‘not at harmful’ and 5 = ‘extremely harmful’.

##### **Perceived addictiveness of e-cigarette use**

Measured by two questions adapted from previous studies [44, 47, 50]. Participants are asked how addictive they perceive daily cigarette and e-cigarette use to be. Response choices are on a 5-point Likert scale, where 1 = ‘not at all addictive’ and 5 = ‘extremely addictive’.

##### **Refusal skills**

Measured by four questions adapted from previous studies [44, 47]. Participants are asked on the ease or difficulty of refusing an e-cigarette offered to them by a friend by using four different strategies (i.e., “avoid the situation”). Response choices are on a 4-point Likert scale, where 1 = ‘very easy’ and 4 = ‘very hard’.

##### **Additional measures**

Gains and soft skills among Year 10 students will be assessed through a post-survey and open-ended questions in the debrief session. In the debrief session, participants are asked what worked well, what was challenging, what they gained as a BEAT Peer Leader and suggestions for improvements.

### Adoption

Adoption will be measured by the number and proportion of schools which elect to participate in the program compared to those approached. Detailed notes will be kept by the research team on recruitment strategies, reasons for non-participation or completion, and school’s implementation methods. Representativeness of schools will be assessed based on key student and school-level factors [51].

### Implementation

Fidelity of workshop and peer education delivery will be assessed using a process evaluation observation table completed by the research team. This table will measure exposure (time taken for activities), adherence (correct use of strategies and resources), and student responsiveness (engagement and interest). Detailed notes will be kept on factors affecting implementation.

Acceptability and usefulness of the program will be assessed through semi-structured interviews conducted with the BEAT program coordinator focusing on Year 10 peer leaders’ gains, aspects of the program which did or did not work well, suggestions for improvement and prior e-cigarette initiatives delivered by the school. Program fidelity and acceptability will also be evaluated during debrief sessions with Year 10 peer leaders.

### Maintenance

Maintenance of the program will be assessed by schools’ willingness to continue implementing BEAT and maintaining a relationship with the research unit. Detailed notes will document any adaptations to the program and changes in schools’ receptivity to the program or research unit.

## Analysis

### Statistical analysis

Data collected will be non-identifiable, cleaned and imported into the R software environment for statistical computing. Participants’ unique ID number will be used to match data from the two time points. Descriptive statistics and changes between data collection points will be assessed for statistical significance (P < 0.05). Wilcoxon signed rank tests (a non-parametric alternative for paired t-tests, pairs are within subject’s pre and post survey responses) will be used for within-subject comparisons. Cluster-robust standard errors will be implemented using the ‘clusrank’ R package [52]. To assess if students who are excluded from analysis (e.g., pre-survey could not be linked to a post-survey using the identifying code) are substantively different from those included in our analysis, we will quantify the comparability between the unlinked and linked participants using standardized mean differences (SMDs) of key pre-test study variables. We will interpret standardized mean differences values of > 0.20 to be large differences between groups. Sensitivity analysis will use multiple imputation to explore the impact of missing data on study results.

### Framework analysis

The qualitative data from questionnaires, year 10 debrief sessions, and observational field notes will be analysed using a Framework Analysis approach. For this study, we will follow the Framework Analysis approach as outlined in Goldsmith and Parkinson et al. ‘s qualitative studies [53, 54]. It is a well-established, systematic, transparent approach known for its practical orientation [55]. Framework analysis includes familiarisation (step 1), framework development (step 2), indexing (step 3), charting (step 4) and mapping and interpretation (step 5). Data will be analysed in alignment with the components of the RE-AIM framework.

## Discussion

This paper describes the study protocol of the BEAT program which has been designed and will be conducted in partnership with schools and the NSW Department of Education. This is the first evaluation of a school-based peer-leadership program to build students’ resilience to e-cigarette use in Australia. Informed by the findings of our formative research, comprehensive consultation with the school community and best practices in e-cigarette prevention, BEAT aims to present a feasible and acceptable solution to the prevention of adolescent e-cigarette use in GWS [25, 56]. A key strength of this novel approach is the utilisation of PERU’s evidence-based peer-education model, which is oriented to social-capacity building and positions students as one of the key drivers of behavioural change within their school community. Additionally, the BEAT program is designed to complement the personal development and health education curriculum and what schools are already doing to address e-cigarette use among students.

While randomised controlled trials have long been considered the gold standard for conducting intervention research [57], it is essential that researchers are flexible to the needs and priorities of schools [58]. In our research context, secondary schools face numerous challenges, including staff shortages, workload burden, and resource constraints which has led to the sidelining of extracurricular activities [59, 60]. To reduce potential burdens associated with rigorous study designs and data collection methods, we have opted to utilise a simple pre-post study design which is acceptable and aligned with the priorities of our partner schools. With appropriate funding and buy-in from schools, we anticipate conducting further studies which incorporate control or comparison groups.

The RE-AIM framework was chosen for its comprehensive approach to evaluating multi-faceted interventions in complex settings, providing insights into both the effectiveness and implementation of the BEAT program. However, our study will be implemented during a period of rapidly evolving legislative change and enforcement which seeks to limit the supply of e-cigarettes in Australia. As e-cigarettes become increasingly scarce and lose saliency within the local community, such advancements may impact schools’ willingness to implement e-cigarette prevention programs over the coming years.

BEAT presents a novel peer-led solution to address a gap in current research practice to prevent adolescent e-cigarette use in Australia. Additionally, it has the potential to protect the future health and wellbeing of students from the harms of e-cigarette use and nicotine addiction. If the program is effective, funding support will be sought to disseminate the program to other regions or countries, and to conduct further evaluation. The program framework and partnerships with schools will additionally establish a foundation from which other substance use and health-related prevention activities may occur in the future. At the conclusion of the study, findings will be prepared for publication in peer-reviewed journals and simplified reports outlining study findings will be shared among participating schools.

## Data Availability

Upon completion of the study, data will be made available upon reasonable request to the study team.

## Abbreviations

ATI-V: Above The Influence of Vaping
BEAT: Breathe Easy All Together
CMB: CATCH My Breath
GWS: Greater western Sydney
ICSEA: Index of Community Socio-Educational Advantage
NSW: New South Wales, Australia
PERU: Prevention Education and Research Unit
PIS: Participant information statement
RE-AIM: Reach, Effectiveness, Adoption Implementation and Maintenance
